# Molecular Modeling Study for Inhibition Mechanism of Human Chymase and Its Application in Inhibitor Design

**DOI:** 10.1371/journal.pone.0062740

**Published:** 2013-04-25

**Authors:** Mahreen Arooj, Songmi Kim, Sugunadevi Sakkiah, Guang Ping Cao, Yuno Lee, Keun Woo Lee

**Affiliations:** Division of Applied Life Science (BK21 Program), Systems and Synthetic Agrobiotech Center (SSAC), Plant Molecular Biology and Biotechnology Research Center (PMBBRC), Research Institute of Natural Science (RINS), Gyeongsang National University (GNU), Jinju, Republic of Korea; University of Edinburgh, United Kingdom

## Abstract

Human chymase catalyzes the hydrolysis of peptide bonds. Three chymase inhibitors with very similar chemical structures but highly different inhibitory profiles towards the hydrolase function of chymase were selected with the aim of elucidating the origin of disparities in their biological activities. As a substrate (angiotensin-I) bound crystal structure is not available, molecular docking was performed to dock the substrate into the active site. Molecular dynamics simulations of chymase complexes with inhibitors and substrate were performed to calculate the binding orientation of inhibitors and substrate as well as to characterize conformational changes in the active site. The results elucidate details of the 3D chymase structure as well as the importance of K40 in hydrolase function. Binding mode analysis showed that substitution of a heavier Cl atom at the phenyl ring of most active inhibitor produced a great deal of variation in its orientation causing the phosphinate group to interact strongly with residue K40. Dynamics simulations revealed the conformational variation in region of V36-F41upon substrate and inhibitor binding induced a shift in the location of K40 thus changing its interactions with them. Chymase complexes with the most activecompound and substrate were used for development of a hybrid pharmacophore model which was applied in databases screening. Finally, hits which bound well at the active site, exhibited key interactions and favorable electronic properties were identified as possible inhibitors for chymase. This study not only elucidates inhibitory mechanism of chymase inhibitors but also provides key structural insights which will aid in the rational design of novel potent inhibitors of the enzyme. In general, the strategy applied in the current study could be a promising computational approach and may be generally applicable to drug design for other enzymes.

## Introduction

**Figure 1:**
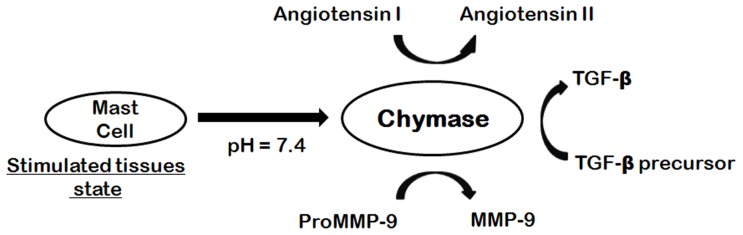
Chymase-dependent conversion of angiotensin I to angiotensin II and precursors of TGF-β and MMP-9 to their active forms.

Chymase (EC 3.4.21.39) is an enzyme of the hydrolase class that catalyzes the hydrolysis of peptide bonds and it is abundant in secretory granules of mast cells. Chymase is the major extravascular source of vasoactive angiotensin II(Ang II), which is generated very efficiently by human chymase via hydrolysis of the Phe-8–His-9 bond of angiotensin I(Ang I) [1]. Chymase is stored in mast cells in an inactive form and is released as an active enzyme when mast cells are stimulated by injury or inflammation. Chymase shows enzymatic activity immediately after its release into the interstitial tissues at pH 7.4 following various stimuli in tissues. As chymase has no enzymatic activity in normal tissues, chymase inhibitors have the potential to be safe/non-toxic because specific chymase inhibitors may not have effects on any other targets in normal tissues [2]. Cardiovascular diseases are the leading cause of death in the developed world and are now on course to emerge as the major cause of death in the developing world [3]. One particular manifestation of cardiovascular diseases, heart failure (HF), is dramatically increasing in frequency. A link between heart failure and chymase has been ascribed, and there is an interest to develop a specific chymase inhibitor as a new therapeutic treatment for the disease [4]. The density of cardiac mast cells is remarkably increased in patients with heart failure, and cardiac chymase may play an important role in the development of several cardiovascular diseases [5]. Recently, it was observed that chymase activation was increased in ischemic myocardium following acute myocardial ischemia/reperfusion (AMI-R) compared to non-ischemic and sham myocardial tissue [6]. Chymase is also known to activate matrix metalloproteinase (MMP)-9 by cleaving a specific site of the catalytic domain of MMP-9. MMP-9, known as 92 kDa gelatinase, is correlated with an increase in infarct sizeand left ventricle (LV) fibrosis following experimental AMI [7]. Chymase also converts the precursor of transforming growth factor-β (TGF-β)to its active form thus contributing to vascular response to injury (Figure 1). Both TGF-β and MMP-9 are involved in tissue inflammation and fibrosis, resulting in organ damage [8]. Previous studies have shown the involvement of chymase in the escalation of dermatitis and chronic inflammation following cardiac and pulmonary fibrosis [9]. Therefore, inhibition of chymase is likely to reveal therapeutic methods for the treatment of cardiovascular diseases, allergic inflammation, and fibrotic disorders. Chymase inhibition may also be useful for preventing the progression of type 2 diabetes, along with the prevention of diabetic retinopathy [10]. Moreover, the role of chymase in inflammation has demonstrated its restorative value in diseases such as chronic obstructive pulmonary disease (COPD) and asthma [11].

Over the past 15–20 years, several peptide and non-peptide inhibitors of chymase have been synthesized [12], [13], [14], [15]. In general, chymase inhibitors readily decompose in plasma, thus the stability of the chymase inhibitors in human plasma has always been a matter of great concern. So, there is a continuing need to search for more stable inhibitors with high activity against human chymase. To date, six crystal structures have been determined for human chymase while four 3D crystal structures of chymase enzyme bound with diverse inhibitors have been determined andare available in the protein data bank (PDB). However, the substrate (Ang I) bound crystal structure has not been solved yet which has obstructed efforts to gain a deeper insight of the structural state of the enzyme’sactive site cleft when accommodating a long peptide substrate.

### Chymase Structure Details

**Figure 2:**
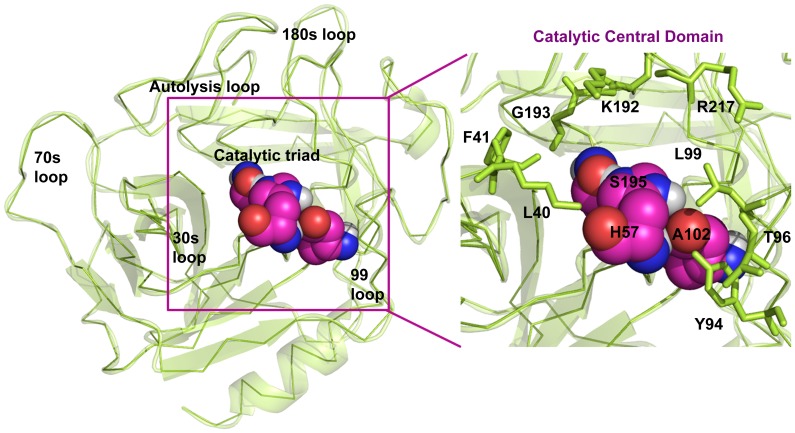
Overall 3D structure of human chymase and the zoomed view which clearly shows the important catalytic residues.

Human chymase is folded into two six-stranded β-barrels, which are connected by three trans-domain segments (Figure 2). Human chymase contains 13 arginine and 15 lysine residues, but only eight glutamate and eight aspartate residues, resulting in a rather high overall basicity. The inhibitor binding region in human chymase is defined by residues K40, H57, Y94, N95, T96, L99, D102, A190, F191, K192, G193, S195, V213, S214, Y215, G216, and R217. This is mostly polar region because of main chain carbonyls that are arranged along the surface of the region. The catalytic residues Ser195, Asp102, and His57 are located at the junction of the two β-barrels, while the active-site cleft runs perpendicular to this junction.

Some evidence exists that the residue at the position 40 has an important role in the enzyme catalysis. Human chymase has been identified as an efficient angiotensin converting enzyme, selectively hydrolyzing Ang I at F8 to generate bioactive Ang II [16]. While, Rat chymase (RMCP-I) degrades the Tyr4-Ile5 bond of Ang-I to inactive fragments. In order to clarify this different catalysis for Ang-I at the atomic level, Yamamoto et al. performed a study which clearly showed the significant difference in the electrostatic potential of the solvent surface [17]. From the modeling study of complex structures with Ang-I, the functional difference between both enzymes was clearly related to the electrostatic difference, especially at the C-terminal substrate-binding site. Site-specific mutagenesis studies have shown that K40 in human chymase influences substrate recognition resulting in cleavage at F8 in Ang I [18]. When K40 was mutated to A, the human enzyme became much less specific for hydrolysis at F8 of Ang whereas Y4 hydrolysis rates(destroys bioactivity of Ang II) were enhanced 16-fold compared to the wild-type human enzyme. Thus, K40 plays a key role in the substrate recognition and hydrolysis based on these studies. A previous study has revealed the substantial variation in terms of human chymase inhibitory activity of very similar chemical compounds [19]. In this study, a strategy that integrates the advantages of molecular dynamics simulations, pharmacophore modeling and molecular docking approaches has been applied, in order to understand the important chemical features that inhibit the chymase enzyme and to determine the molecular mechanism by which these compounds act. Molecular docking is applied to prepare the enzyme-substrate and enzyme-inhibitor complexes when their crystal structures are not available. A hybrid pharmacophore model was also developed using MD refined enzyme-substrate and enzyme-inhibitor complexes. Interesting and valuable information has been obtained from the analysis of binding modes of inhibitors, structural changes at the active site, and interaction energy computations which effectively explained the differences in the activity of inhibitors. In summary, the findings from this research could be helpful to design and identify potentially useful novel chymase inhibitors for the treatment of cardiovascular diseases, allergic inflammation, and fibrotic disorders.

## Materials and Methods

### Selection of Inhibitors and Preparation of Complex Structures

**Figure 3:**
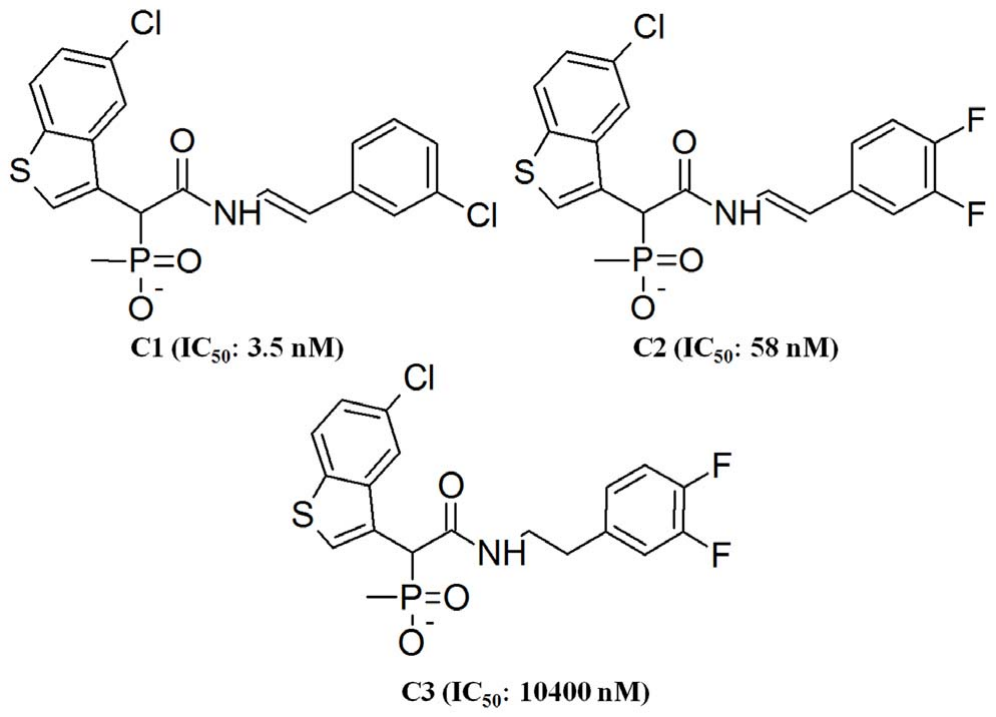
The 2D chemical structures of human chymase inhibitors used in this study along with their IC_50_values.

Three chemical compounds with their experimentally known chymase inhibitory activity (IC_50_) data were obtained from the literature. These compounds, labeled as C1, C2, and C3 havevery similar chemical structures but they exhibit considerable differences in terms of chymase inhibitory activity (Figure 3) [19]. With the aim of examining the structural changes to address the observed differences in their inhibitory activity, chymase inhibitor complexes were prepared for MD simulations. The crystal structure of chymase complexed with C2 (PDB code 3N7O) was already available in PDB and it was directly used in MD simulations [20]. However, crystal structures of C1and C2 are not available. Therefore, the crystal structure of chymase bound with inhibitor C2, which has very similar structural profile with C1 and C3 was utilized in preparing their complexes. In order to prepare a complex structure of the most active compound C1, the similar compound present in 3N7O was modified by replacing one of the fluorine atoms attached to the phenyl group with chlorine while the other fluorine atom was removed. The carbon-carbon double bond of C2 was modified to a single bond to prepare the complex structure of the least active compound C3. Ang I is the natural substrate of chymase but the substrate (Ang I) bound crystal structure has not been solved yet. Crystal structures of chymase-MMP-9 and chymase TGF-beta complexes are also not available. Due to the large amount computational calculations required for each complex, extending the current study to include these two chymase complexes is not possible. Separate studies will be performed for chymase-MMP-9 and chymase TGF-beta complexes in near future. In order to prepare complex structure of chymase-Ang I, the chemical structure of Ang I was downloaded from PDB(PDB code 1N9U) and docked into the active site of the average structure prepared from a 5 ns MD simulation of chymase apoform. In this study, the molecular docking methodology using *HADDOCK*(High Ambiguity Driven protein−protein Docking) was utilized to obtain the reliable binding mode of Ang I [21].

### Molecular Dynamics Simulations

Biological systems are dynamic in nature; analyzing their motion at the molecular and atomic level is therefore essential to understand their profound dynamic mechanisms such as switching between active and inactive states, intercalation of drugs into DNA, and assembly of microtubules, by studying their internal motions [22], [23], [24]. Therefore, to really understand the interaction mechanism of a receptor with its ligand, investigations should be made not only to their static structures but also of the dynamic process obtained by simulating their internal motions. Molecular dynamics simulation stands alone as the fundamental computational tool for capturing dynamic events of scientific interest and pharmaceutical relevance. To perform calculations, the initial coordinates for the protein atoms were taken from the complex structures of chymase-inhibitor, chymase-substrate complex, and apoform. The protonation states of all ionizable residues were set to their normal states at pH 7. Five MD simulations were performed for systems including the apoform, inhibitors and substrate complexes. All MD simulations were performed with GROMOS96 forcefield using GROMACS 4.5.3 package running on a high performance linux cluster computer [25], [26]. During MD simulations, all the protein atoms were surrounded by acubic water box of SPC3 water molecules that extended 10 Å from the protein and periodic boundary conditions were applied. The systems were neutralized with Cl^−^ counter ions replacing the water molecules and energy minimization was performed using steepest descentalgorithm for 10,000 steps. A 100 ps position restrained MD simulation was performed for every system followed by a 5 ns production MD simulation with a time step of 2 fs at constant pressure (1 atm) and temperature (300 K). The electrostatic interactions were calculated by the PME algorithm and all bonds were constrained using LINCS algorithm. A twin range cutoff was used for long-range interactions including 9 Å for van der Waals and 14 Å for electrostatic interactions. The snapshots were collected at every 1 ps and stored for further analyses of MD simulations. The system stability and behavior of the catalytic structural components present in every system were analyzed using the tools available with GROMACS 4.5.3 and PyMol programs.

### Development of Hybrid Pharmacophore Model

The hybrid 3D pharmacophore hypotheses were developed using representative structures collected from the MD simulations of C1 and Ang I complexes, and based on features identified from C1 (the most active compound) and Ang I the substrate. The *Receptor-Ligand Pharmacophore Generation* protocol of Accelrys Discovery Studio v3.0 (DS), Accelrys, San Diego, USA, was applied to accomplish this task with default parameters. This protocol generates selective pharmacophore models based on receptor-ligand interactions. An investigation based on the importance of all generated pharmacophoric features was performed to select the features to complement the very important interaction points at the active site. Finally, the *Merge pharmacophore* tool of DS was used to merge key features for the formation of a hybrid pharmacophore model. Utilization of this hybrid pharmacophore model development methodology is wholly new and different methodology from the common feature and structure-activity relationship based pharmacophore models employed in our previous study for designing human chymase inhibitors [27].

### Druglike Database Screening and Molecular Docking

Maybridge, and Chembrige, commercial chemical databases containing 59 652, and 50 000 compounds, respectively, were employed for a virtual screening procedure. However, these databases contain a number of nondruglike compounds. As, it is worthless to screen all the compounds of these databases and then eliminate them in the later phase for their nondruglike properties, the compounds not exhibiting druglike properties were excluded from the databases prior to hybrid pharmacophore-based virtual screening. In order to accomplish this task, compounds in these databases were subjected to various filters for druglike structure, such as Lipinski’s rule of five and ADMET(absorption, distribution, metabolism, excretion, and toxicity) properties [28]. *Prepare Ligands* and *ADMET Descriptors* protocols as available in the DS program were used in this step. After preparation of druglike databases, the hybrid pharmacophore model was employed for screening of these druglike databases to select the compounds containing the hybrid pharmacophoric features identified from both the inhibitor and the substrate. The druglike hit compounds that were identified to have hybrid pharmacophoric features were subjected to molecular docking studies using GOLD (Genetic Optimization for Ligand Docking). The *Prepare Ligands* protocol as implemented in DS was employed to change the ionization states as well as to generate different tautomers and isomers of the hit compounds. GOLD 5.1 program from Cambridge Crystallographic Data Center, UK. uses a genetic algorithm for docking ligands into protein binding sites to explore the full range of ligand conformational flexibility with partial flexibility of protein [29], [30]. Protein coordinates from the representative structure of the chymase-C1 complex obtained from MD simulation were used to define the active site. The active site was defined with a 10 Å radius around the bound inhibitor. Ten docking runs were performed per structure unless five of the ten poses were within an RMS deviation value of 1.5 Å. The GOLD fitness score is calculated from the contributions of hydrogen bond and van der Waals interactions between the protein and ligand, intramolecular hydrogen bonds and the internal strain of the ligand. Protein-ligand interactions were analyzed using DS. For further validation, electronic parameters and binding free energies were calculated for all the known active compounds and database hits using *Gaussian 09*and AutoDock 4.2 programs, respectively [31], [32]. The final hit compounds with the best electronic parameters and binding energies were selected.

## Results and Discussion

### Selection of Chymase Inhibitors

Three chymase inhibitors used in this study are very similar in terms of their chemical structures but their human chymase inhibitory activity (IC50 values) values tested using the same biological assay were extremely different from one another (Figure 3). The most active C1 holds the same basic scaffold as other compounds in the study but the observed IC50 value is 17 fold higher for C1 compared to C2. It contains a chlorine atom attached to the aromatic ring instead of two fluorine atoms for compounds C2 and C3. Furthermore, the only difference between the structures of C2 and C3 is the presence of a double bond between carbon atoms in compound C2. However, in spite of these small differences, the IC50 value of C2 is 58 nM,180 fold more potent than its counterpart C3 (10 400 nM). Alternatively, absence of a double bond in C3 has lowered 180 fold its IC50 value. This drastic change in the human chymase inhibitory profiles of these very similar chemical compounds has triggered our interest and motivated us to study the structural reasons which may possibly explain this behavior. The recognition of structural responses to these structurally very similar inhibitors with different inhibitory activities can be employed in designing highly potent novel drug candidate for further experimental investigation. It may also aid to develop new and effective drug for the treatment of cardiovascular diseases, allergic inflammation, and fibrotic disorders.

### Overall Stability of the Systems

**Figure 4:**
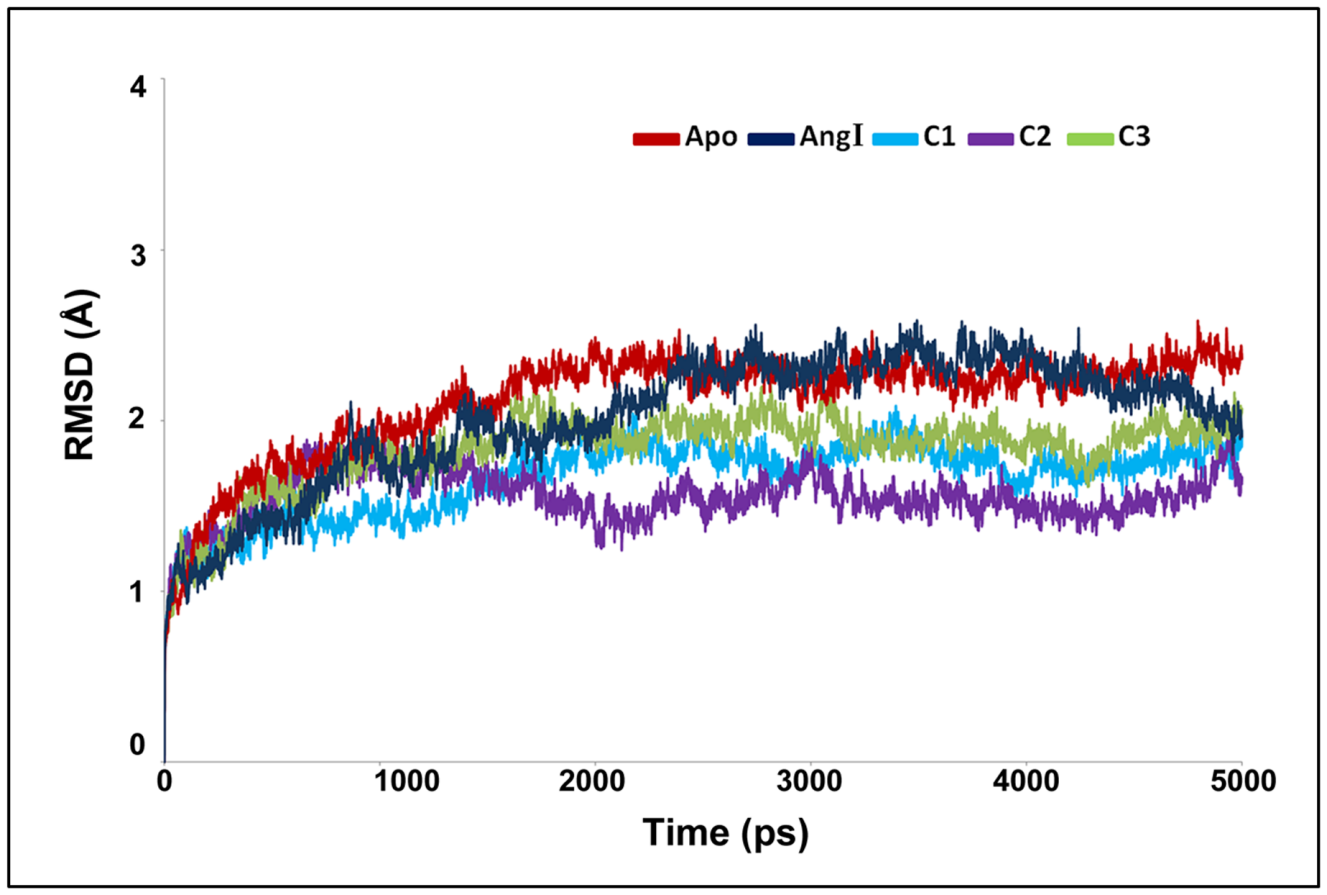
The RMSD plot to investigate the stability of the systems.

**Figure 5:**
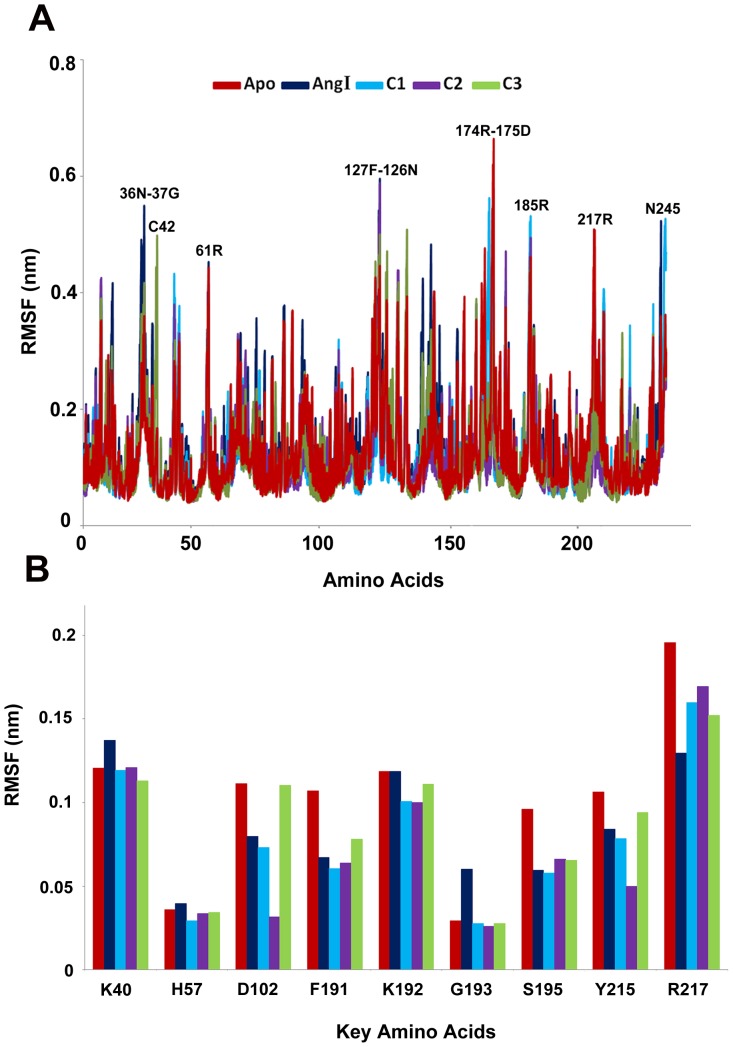
The RMSF plots of all systems (A) full protein and (B) important active site residues.

MD simulation results of all the systems were used for examining the stability of every system. The root mean squaredeviation (RMSD) from initial conformation is a central criterion used to evaluate the difference of the protein system. The stabilityof each simulation system was evaluated based on its RMSD (Figure 4). RMSD of the chymase-apoform displayed the highest value compared to all other systems. While, the RMSD of the complex with C2 inhibitor showed the lowest value among other inhibitors. Though differences were observed in RMSD values among the systems, they all converged to around 2.0 Å except apoform and the Ang I-complex which had a mean value slightly over 2.0 Å. The higher RMSD value of the Ang I-complex (2.01 Å) depicts the structural flexibility of the system upon binding of Ang I when compared to others. Comparison of the mean RMSD values of C1and C2, which are 1.66 Å and 1.55 Å, respectively, shows that the flexibility observed in the structure is caused by the replacement of fluorine with chlorine atom in the structure. The calculated mean RMSD values of C2 and C3 were 1.55 Å and 1.82 Å, respectively, which showed the influence of conversion of double bond to single bond in C3. Interestingly, all complexes have shown the RMSD values smaller than that of apoform explaining the difference in terms of instability in chymase complexes. The root mean square fluctuation (RMSF) values of the systems were also calculated and plotted to compare the flexibility of each amino acid residues of the complex. This RMSF plot reveals the flexible regions of the systems during the simulation time (Figure 5A). Moreover, in order to investigate the motions about the important residues interacted with the inhibitor in the binding site; the root mean square fluctuations (RMSF) for all important catalytic residues were calculated and depicted in Figure 5B. This focused RMSF plot clearly shows that none of the important catalytic residues present in the active site has an RMSF value more than 2Å (Figure 5B). This result has confirmed that the catalytic machinery present in the active site was minimally distorted upon binding of substrate and any of the inhibitors. The amino acids, namely, K40, K192, and R217 have shown the flexibility upon the binding of different ligands. Most of these residues were highly mobile in the apoform of the enzyme whereas they were stable in complex systems indicating the binding nature of the ligands (Figure 5B). In case of C3, and chymase-Ang I complexes, the majority of the active site residues exhibited more fluctuation as compared to other systems. Particularly, R217 fluctuated more compared to other amino acids in the active site indicating its flexible nature in apoform and complex systems.

### Mode of Substrate Binding in Human Chymase

**Figure 6:**
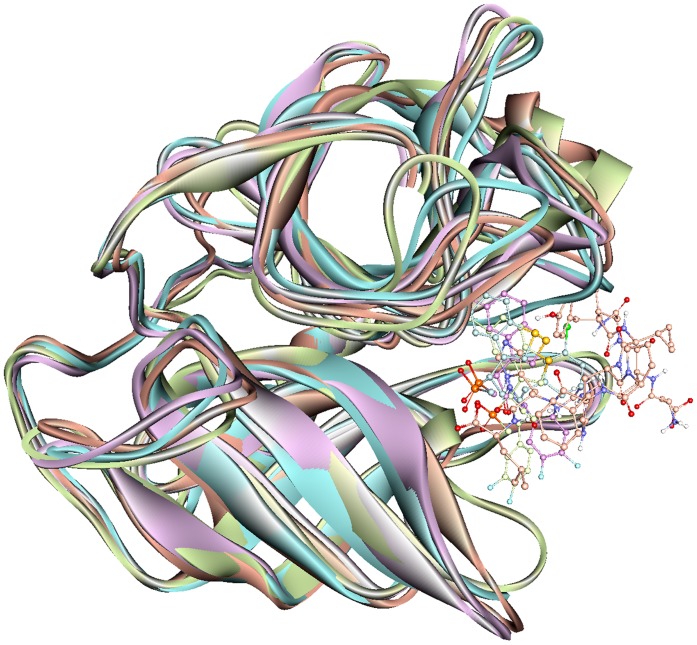
Overlay of the binding modes of the substrate and all the inhibitors at the active site of human chymase enzyme. The Ang I, C1, C2, and C3 complexes were represented in orange, cyan, violet, and splitpea green colors, respectively.

**Figure 7:**
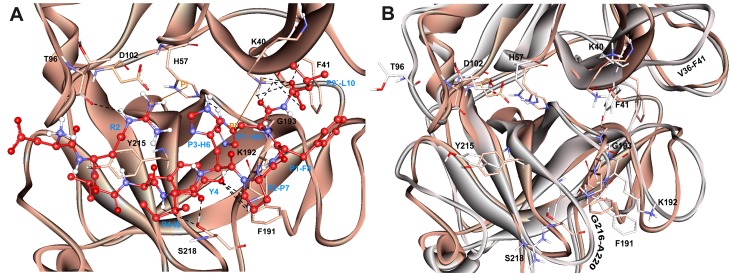
Active site of the substrate (Ang I) complex of the enzyme. (A) Amino acid residues and bound substrate are shown in thin stick and ball-stick forms, respectively. Residues of substrate are shown in red color and labeled in blue color. Hydrogen bonds are shown in black dashed lines. (B) The structural changes observed between the active site regions of apoform (grey) and human chymase-substrate complex (orange). The amino acid residues are shown in thin stick form. Only polar hydrogen atoms are added for clarity.

**Figure 8:**
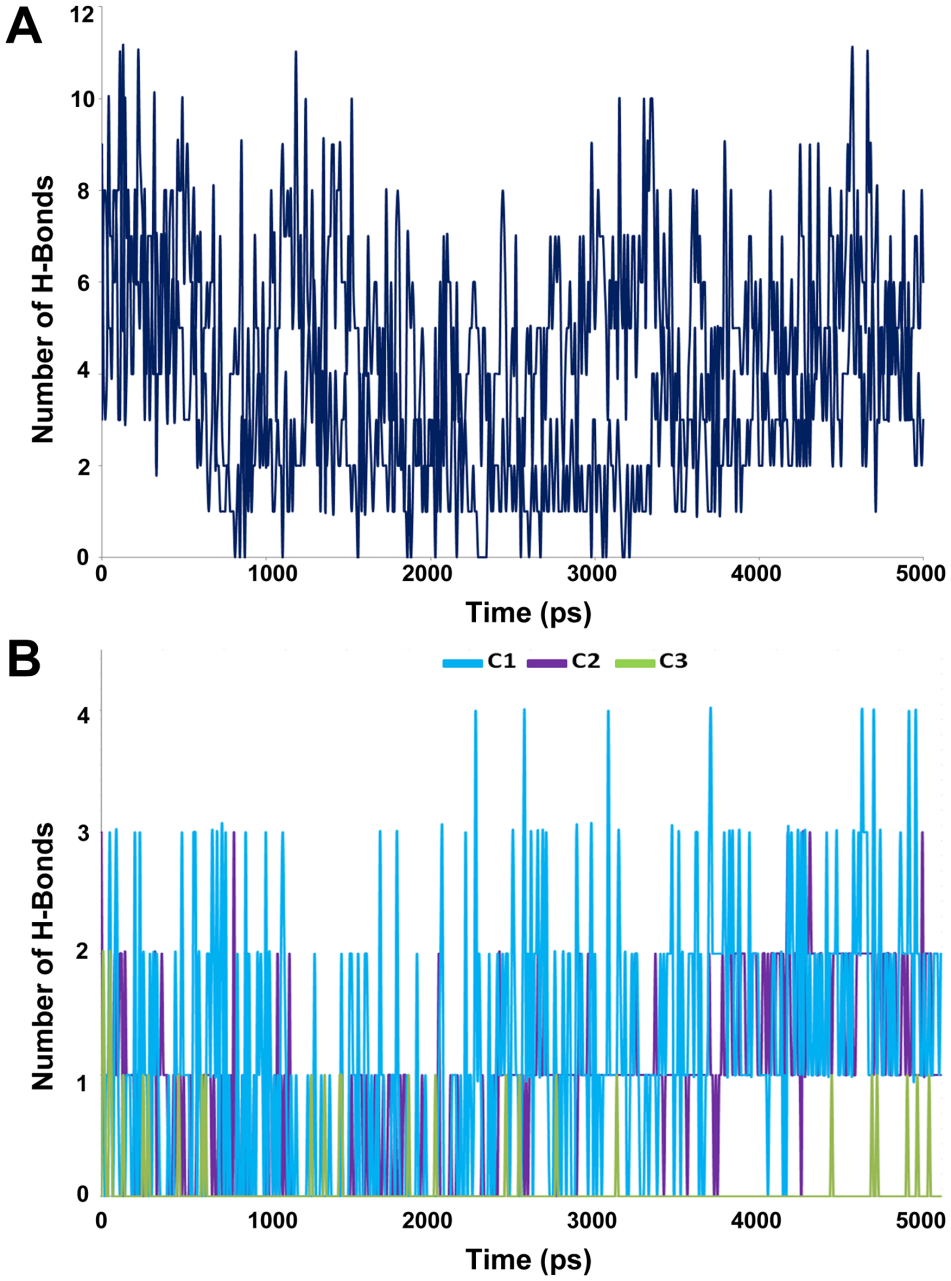
Intermolecular hydrogen bond plots. Observed intermolecular hydrogen bonds between (A) the substrate (B) inhibitors and active site residues.

The representative structure of the apoform of the enzyme selected from the MD simulation was employed to dock the substrate Ang I into the active site cleft of chymase enzyme. In view of the fact that the crystal structure of the enzyme-substrate complex has not been determined yet, the proposed binding mode of the substrate is very important to understand the hydrolase mechanism of the enzyme. Some previous studies have also proposed its binding mode at the active site of the enzyme. In chymase, as in other trypsin-family serine peptidases, key substrate binding interactions occur in an active site cleft formed between two, six-stranded β-barrel domains [33]. The nomenclature of Schechter and Berger (1967) is adopted to designate the substrate residues as P2, P1, P1′, etc., for substrate binding mode analysis, where P1 is the amino acid after which the peptide bond is cleaved. In the case of chymase-mediated hydrolysis of Ang I, interactions involving P1, P1′, and P2′ are particularly important. All the ligands under study bind the same active site pocket but they vary in terms of their molecular interactions (Figure 6). The proposed binding mode of Ang I is that P1′-H9 is positioned in such a way to cause close hydrogen bond contacts(2.489, 2.623 Å) with the positively charged side chain of K40 residue from a complementary binding site S1′ thus enabling the catalysis (Figure 7A). Moreover, a π^…^cationic interaction was also observed between P1′ and K40. In addition, a hydrogen-bonding network (1.633, 3.036, 3.131, and 3.280 Å) between the P2′-L10 side chain and K40 is also established which we suggest stabilizes the Ang I extended into the protein from this point. P1′-H9 of Ang I threads through the active site S1′ pocket and form a hydrogen bond with H57 of chymase. F41 of chymase is proximal to the P2′ side chain thus providing the potential for a hydrogen bond to be formed with the carboxylate group of P2′-L10. The P4-I5 of AngI is also able to make hydrogen bonds with the polar S218 residue of the enzyme. The other residues of the binding site cleft such as T96, and F191 have also formed hydrogen bonds with the substrate (Figure 7A). The binding mode of Ang I at the active site has formed a number of hydrogen bonds with the active site residues throughout the simulation time indicating its strong binding affinity (Figure 8A). The structural analysis of the active site upon binding of substrate indicated that the catalytic residues in substrate complex were very stable compared to the active site of apoform except for small side chain movements that lead the key residues like K40, H57, D102, F191, Y215, and S218 close to the substrate for stronger molecular interactions (Figure 7B). Significant structural changes were observed in and around the active site thus impacting the binding mode of critical residues to the substrate. Particularly, the change in the loop region formed by V36-F41 has caused a critical conformational variation upon the important K40 residue. Thus, K40 is ideally positioned to form putative interactions with the key residues P1′-H9 and P2′-L10 of substrate. Moreover, change in the conformation of the G216-A220 loop region instigated a significant modification in position of the S218 residue of the binding site cleft. These binding characteristics observed from the Ang I-substrate simulation are compared with the binding modesof inhibitors.

### Binding mode Analysis of Chymase-inhibitor Complexes

#### Chymase-C1 complex

**Figure 9:**
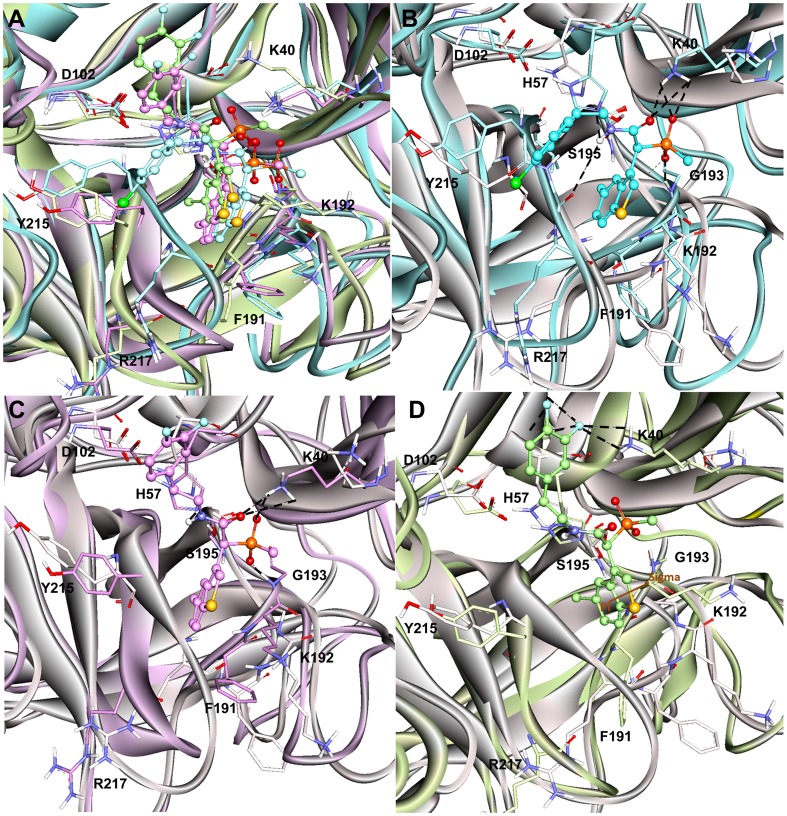
Binding modes and molecular interactions of the three inhibitors. (A) Overlay of the binding modes of all the inhibitors at the active site of human chymase enzyme. (B) C1 in cyan (C) C2 in violet (D) C3 in splitpea green colors at the active site of enzyme. The hydrogen bond and π-interactions were displayed in black dashed and brown solid lines, respectively. Amino acid residues and inhibitors are shown in stick and ball-stick forms whereas the gray, cyan, violet, and splitpea green cartoons represent apoform of enzyme, C13, C14, and C15 complexes, respectively. Only polar hydrogen atoms are added for clarity.

C1 is the most active chymase inhibitor among the compounds under study which possess a chlorine atom attached to the aromatic ring instead of two fluorine atoms as in case of C2 and C3 compounds. The binding mode of C1 at the active site has formed maximum number of hydrogen bonds with the active site residues throughout the simulation time indicating its excellent binding affinity as compared to C2 and C3 inhibitors (Figure 8B). The phosphinate group of C1 drove the inhibitor to form closer interactions with the key residues K40 (2.418, 2.904 Å) and G193 (1.806 Å) of active site region (Figure 9B). The oxygen atom of amide carbonyl group is also involved in the formation of hydrogen bonds contacts with K40(3.021, 3.214Å). Compound C1 also interacted with the polar residue H57 (3.025 Å) through amide moiety. In terms of active site flexibility upon binding of C1, the positions of key residues are changed as compared to apoform of enzyme. The polar residue S195 of catalytic triad has moved away to accommodate C1 at the active site cleft. While, the charged residues K40 and R217 along with H57 have moved closer to the inhibitor to form stronger interactions with it, thus stabilizing the binding conformationof C1at the active site region. The most important change in the active site pocket upon binding of C1 is the variation in position of K40. The loop region consisting of V36-F41 residues has moved into the active site cleft thus changing the orientation of positively charged K40 residue closer to the bound inhibitor. The same loop region also fluctuated for substrate binding mode but it showed less variation as compared to the loop fluctuation during C1 binding to the active site of enzyme.

#### Chymase-C2 complex

The binding mode of C2, the mid-active compound, also formed interactions with the key residues inside the active site of the enzyme (Figure 9C). As compared to C1, the placement of lighter halide F atoms instead of heavier Cl atom at the phenyl ring in C2 has caused significant variation in its orientation through the displacement of 3,4-difluorophenyl group much away from the benzothiophene moiety. The phosphinate group of C2 compound is also involved in hydrogen bond interactions with K40 and G193 like C1 compound. Both oxygen atoms of the phosphinate have formed hydrogen bond contacts with the positively charged K40 (3.144, 3.212, and 2.012 Å) and G193 (1.992 Å). Moreover, amide group has formed a hydrogen bond interaction with H57 (2.746Å). Although, the C2 inhibitor has exhibited interactions with the key residues, these interactions are relatively long-ranged and less in number as compared to C1. As far as structural flexibility of active site residues is concerned, much variation was observed upon binding of C2 in the active site region. Although, the same loop region consisting of V36-F41 residues has also moved into the active site region as it did upon binding of substrate and C1 inhibitor. However, the fluctuation of this loop was much less as compared to the chymase complex structures of substrate and C1 inhibitor. The binding mode of C2 at the active site has also formed anumber of hydrogen bonds with the active site residues throughout the simulation time showing its strong binding affinity (Figure 8B).

#### Chymase-C3 complex

C3 is the least active chymase inhibitor among the compounds under study and it is the saturated analogue of C2. The conversion of one double bond between two carbon atoms of C2 inhibitor into single bond in C3 has brought drastic change in its potency thus making it 180-fold less potent as compared to C2. Analysis of C3 binding mode revealed that this slight change in its structure had a great impact on its binding conformation in the active site pocket of enzyme (Figure 9D). The presence of single bond adjacent to amide group made the 3,4-difluorophenyl group more able to move thus allowing it closer to the 30 s loop. This flexible movement of the 3,4-difluorophenyl group leads to the benzothiophene moiety becoming tilted further away from the catalytically very important K40 residue of active site. Thus, the phosphinate group of C3 was unable to generate any kind of interactions with K40. C3 did not exhibit any interactions with key residues like H57, G193. The F atom of 3,4-difluorophenyl group formed two relatively long-ranged hydrogen bonds (3.312 Å, 3.327 Å) with K40. Significant structural changes were observed in and around the activesite upon binding of C3 which influenced the locations of critical residues at the active site region. Due to the absence of intermolecular interactions, the variable region of V36-F41 has been very flexible and has moved far away from the original conformation of this loop present in the apoform of enzyme. This movement restrained the phosphinate group of C3 to make any contacts with the key residues like K40. The binding mode of C3 at the active site has also displayed minimum number of hydrogen bonds with the active site residues throughout the simulation time showing its least binding affinity as compared to C1 and C2 (Figure 8B).

### Energetics of Chymase-ligand Complexes

The interaction energy which is defined as the sum of the van der Waals and electrostatic energy contributions to the non-bonded interaction energies of all the ligands was calculated using *Calculate Interaction Energy* protocol implemented in DS. The total calculated interaction energies were −141.51, −106.37, −110.39, and −179.04 for Ang I, C1, C2, and C3 complexes, respectively. These interaction energy values correlate well with the experimental IC50 values where C1, the most active compound, has exhibited better interaction energy than other compounds. The vdw energy of this compound is higher than all other compounds and it was not compensated by the electrostatic energy. All other complexes have also demonstrated favorable vdw interaction energies however these positive vdw interaction energies were compensated by electrostatic interaction energy in particular for chymase systems complexed with C3 and substrate. A careful analysis of the non-bonded interaction energy contribution by active site residues has revealed that some residues showed substantial effects over ligand binding (Table 1). The positively charged K40 residue that interacts with the phosphinate group of C1 and C2 compounds also exhibited interactions with the 3,4-difluorophenyl moiety of C3 along with P1′ and P2′ of substrate, and has shown major contributions to the electrostatic energy in the ligand binding. The polar H57 residue has shown positive electrostatic interaction energies in all the complexes except C1 complex suggesting the importance of this particular residue in contributing to the biological response. In case of F41, which is present in the region where the carboxylate group of P2′-L10 of substrate binds in the active site, all the complexes have shown less contribution from vdw terms, however, their electrostatic energies have contributed to the interaction energies. The C1 and C2 compounds have shown favorable interaction energies with F191, G193, and S195 when compared to the C3 ligand. These interaction energy explorations for the ligands under study have unveiled the information regarding the extent of participation of active site residues and ligands impacting the interactions at the binding cleft of enzyme.

**Table 1:** Calculated non-bonded interaction energies between the inhibitors and important active site residues.

Residue	AngI		C1		C2		C3	
	vdw	Elec	vdw	Elec	vdw	Elec	vdw	Elec
**K40**	−1.18	−110.52	2.12	−110.45	−1.95	−101.44	−2.215	−94.74
**F41**	−1.25	5.11	−0.61	7.88	−0.48	5.14	−0.17	11.31
**H57**	−1.55	5.54	−4.91	−0.13	−3.28	6.20	−1.65	3.86
**D102**	−0.07	−29.07	−0.24	30.67	−0.38	39.82	−0.40	40.97
**F191**	−0.74	−7.44	−3.09	0.73	−1.58	2.18	−2.40	−14.72
**K192**	1.56	−44.52	−4.82	−37.34	−5.14	−53.05	−2.12	−52.93
**G193**	−0.57	2.28	1.60	−11.74	0.01	−7.41	−0.15	−2.40
**S195**	−0.30	2.55	−0.52	7.26	−2.01	8.21	2.63	−2.08
**Y215**	−4.64	8.15	4.87	−4.32	−1.33	−10.9	−1.80	−1.61
**R217**	−1.27	−5.80	−2.57	−0.17	−0.59	−0.032	−0.04	−1.09

### Development of Hybrid Pharmacophore Model

**Figure 10:**
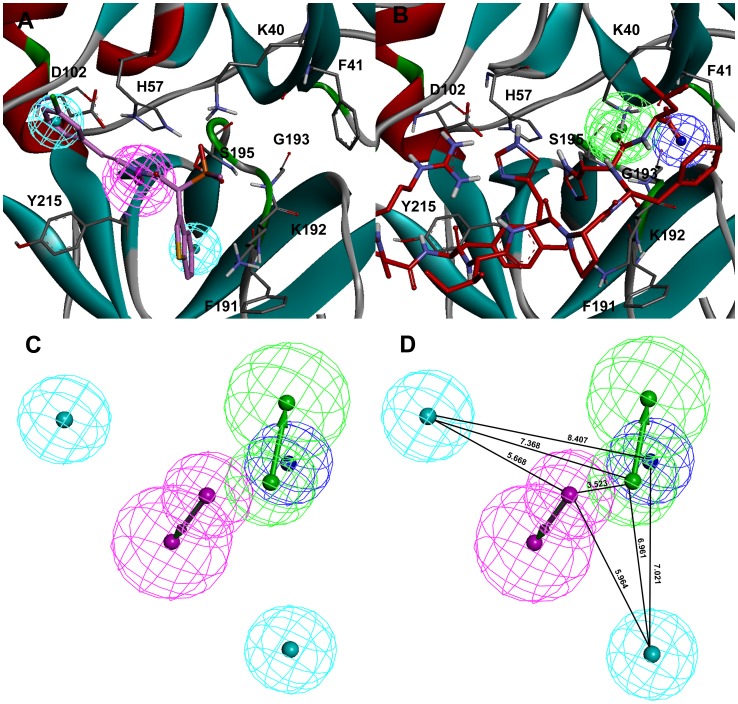
Development of hybrid pharmacophore model. The key pharmacophoric features generated from the binding modes of (A) C1 and (B) Ang I. The amino acid residues of the enzyme are shown in gray thin stick form whereas the bound C1 and Ang I are shown in thick stick form. The secondary structure cartoon of the enzyme is colored based on the hydrophobicity of the amino acid residues. (C) Final hybrid pharmacophore model (D) Hybrid pharmacophore model with distance constraints.

The *Receptor-Ligand Pharmacophore Generation* protocol of DS presents the chemical features which instigate key interactions between the protein and ligand. In this study, the representative structure obtained from MD simulation trajectories of Chymase-C1 complex was used as input for structure-based pharmacophore generation. The *Feature Mapping* protocol has generated all the pharmacophoric features present in the bound conformation of this most active compound at the active site of chymase. The generated pharmacophore model includes three hydrophobic (HY), one hydrogen bond donor (HBD) and one negative ionizable (NI) features. This initial pharmacophore model has two overlapping HY features on the chlorophenyl ring while the third HY feature is overlaid on the benzothiophene moiety of the inhibitor. The HBD and NI features are placed over the amide and phosphinate groups of the bound inhibitor. In the final pharmacophore model generated form Chymase-C1 complex, the overlapping HY feature was removed to simplify the model (Figure 10A). The representative structure obtained from the MD simulation of Chymase-Ang I complex was also used as input for structure-based pharmacophore generation. The initial pharmacophore model contained five features including three hydrogen bond acceptors (HBA), one HBD, and one NI features. Among these five pharmacophoric features, one HBA and NI features are most important due to their location in the binding cleft of enzyme. One of the HBA feature is overlaid on the P1′ residue of Ang I substrate. This feature is oriented towards the positively charged K40 residue of active site. Both, K40 and F41 residues are reported to position the substrate at the active site for the effective catalysis [18]. The presence of negative ionizable (NI) or hydrogen bond acceptor (HBA) groups instead of PI or HBD groups can improvethe binding of ligand by forming strong molecular interactions with the positively charged K40 residue (Figure 10B). The pharmacophore developed from the C1 binding mode also contains a NI feature close to the K40 residue. Therefore, the overlapped NI feature of pharmacophore generated from chymase-C1 complex was deleted. Hence, a final hybrid pharmacophore model containing one HBD, two HY features generated from the binding mode of most active compound (C1) and one NI and one HBA feature generated from Ang I binding mode was developed (Figure 10C). This pharmacophore model was generated in order to screen the chemical compounds with NI and HBA feature as present in Ang I.

### Comparison with the Models of Literature

Our hybrid pharmacophore model is in agreement with the information provided by the former studies. The importance of hydrophobic features is confirmed for chymase inhibition [27], [30], [34]. Moreover, the presence of hydrogen bond acceptor features is also considered significant for inhibition of chymase enzyme [35]. In addition, the methodology to develop this hybrid pharmacophore model development is completely new and different from the common feature and structure-activity relationship based pharmacophore models employed in our previous studies for designing human chymase inhibitors [27].

### Druglike Database Screening and Molecular Docking

Virtual screening is emerging as a productive and cost-effective technology in rational drug design for the identification of novel lead compounds from large virtual database. The final hybrid pharmacophore model was employed as 3D structural query in screening drug-like databases including Maybridge, and Chembrige. From the virtual screening process, 127 and 28 compounds mapping all the five features of the hybrid pharmacophore model were retrieved from Maybridge and Chembrige druglike databases, respectively. Finally, a total of 155 compounds satisfying all the filters was selected and subjected to the molecular docking calculations using *GOLD* program along with the three experimentally known chymase inhibitors.

**Figure 11:**
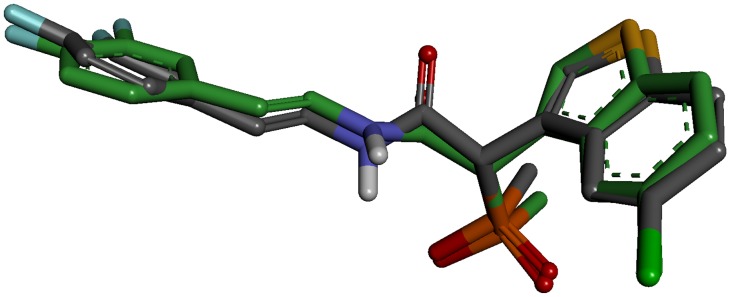
Overlay of the docked pose (green) of inhibitor with its crystal structure conformation (gray).

An initial validation of the docking protocol is performed by comparing the conformation, position, and orientation (the pose) of a ligand as obtained from docking with the one determined experimentally with X-ray crystallography. Correctly redocking the crystallographically observed inhibitor is a minimum requirement to determine whether the program is applicable to this system or not. Crystal structure with the PDB code 3N7O bound with an inhibitor molecule (N7O) was selected as receptor and the active site was defined with a 10 Å radius around the ligand present in the crystal structure. The top conformation of ligand predicted by GOLD program was very close to the crystal structure-bound conformation. The RMSD between the docked pose and its bound conformation in the crystal structure is 0.53 Å indicating that GOLD is able to reproduce correct pose (Figure 11).

In order to find out whether there were interactions between the key residues of the active site of enzyme and the docked compounds, the docking experiment served as a snapshot of molecular interactions The binding modes of all the docked compounds were analyzed for their molecular interactions at the active site. The most active compound C1 in the training set has scored the GOLD fitness score of 67.944. While, among 155 hit compounds, 21 hits demonstrated higher *GOLD* fitness scores thanC1, and were selected for further study.

**Figure 12:**
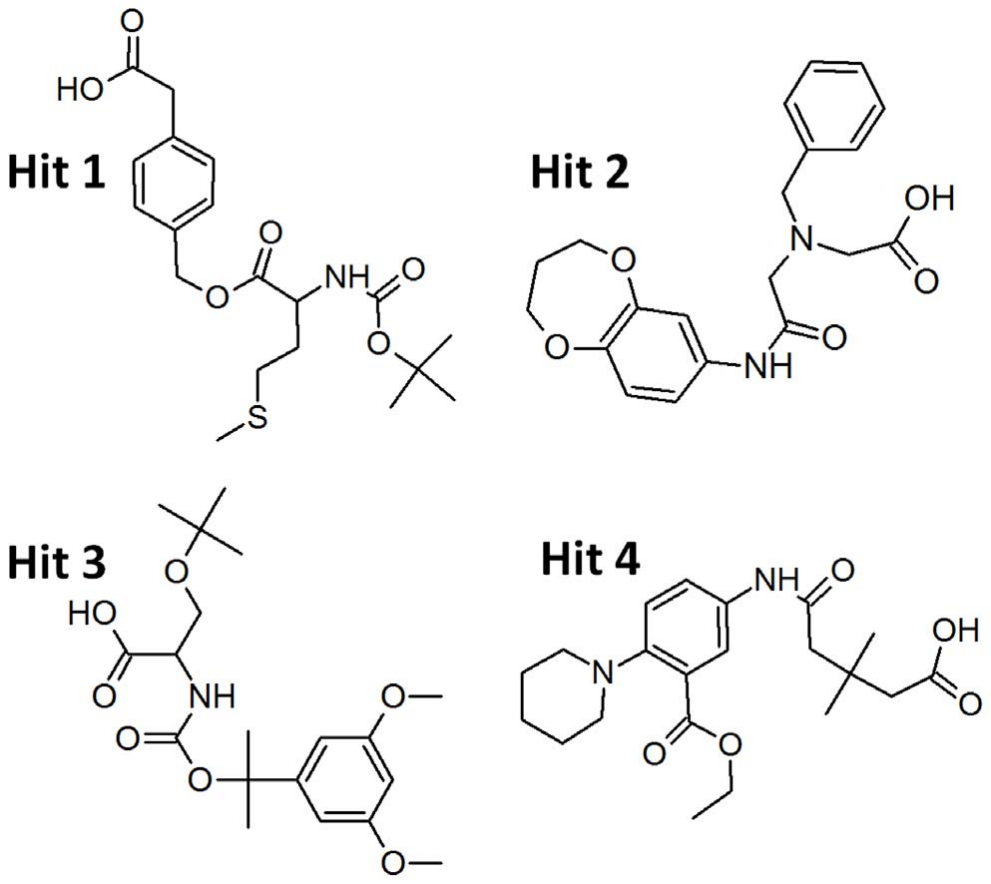
The 2D chemical structures of the final identified hit compounds.

The binding modes of the possible inhibitors for human chymase were further analyzed using Autodock 4.2. This program takes more time but predicts the binding conformations more accurately and results in the binding energy of each docked compound. As an efficient post-docking filtering protocol, the electronic parameters such as highest occupied molecular orbital (HOMO), least unoccupied molecular orbital (LUMO), and energy gap(ΔE) values along with the binding free energies were also calculated for all the compounds. Finally, four compounds were listed as possible chymase inhibitors on the basis of strong binding interaction at active site of target protein, good GOLD Fitness score, and favorable Autodock binding free energy along with highly reactive electronic properties, (Figure 12). The GOLD fitness score and the calculated binding energy values along with the electronic parameters for all the final hitsare given in Table 2. The binding modes and molecularinteractions between the final hits and theactive site components of the enzyme are discussedbelow.

**Table 2:** Results of molecular docking using GOLD and Autodock programs along with electronic parameters calculated for the known inhibitors and hit compounds.

Name	Ligand	GOLDfitness	Binding freeenergy kcal/mol	HOMO (eV)	LUMO(eV)	Energy gap(eV)
C1	C1	67.944	−6.74	−0.100	−0.082	0.018
C2	C2	62.581	−5.51	−0.206	−0.144	0.062
C3	C3	51.236	−5.07	−0.117	−0.083	0.034
Hit 1	JFD_01598	72.018	−6.42	−0.189	−0.093	0.096
Hit 2	SCR_00479	70.268	−5.72	−0.254	−0.184	0.070
Hit 3	RJC_02222	70.107	−6.21	−0.135	−0.127	0.008
Hit 4	HTS_02685	68.195	−5.11	−0.279	−0.250	0.029

### Binding Mode of Hit 1

**Figure 13:**
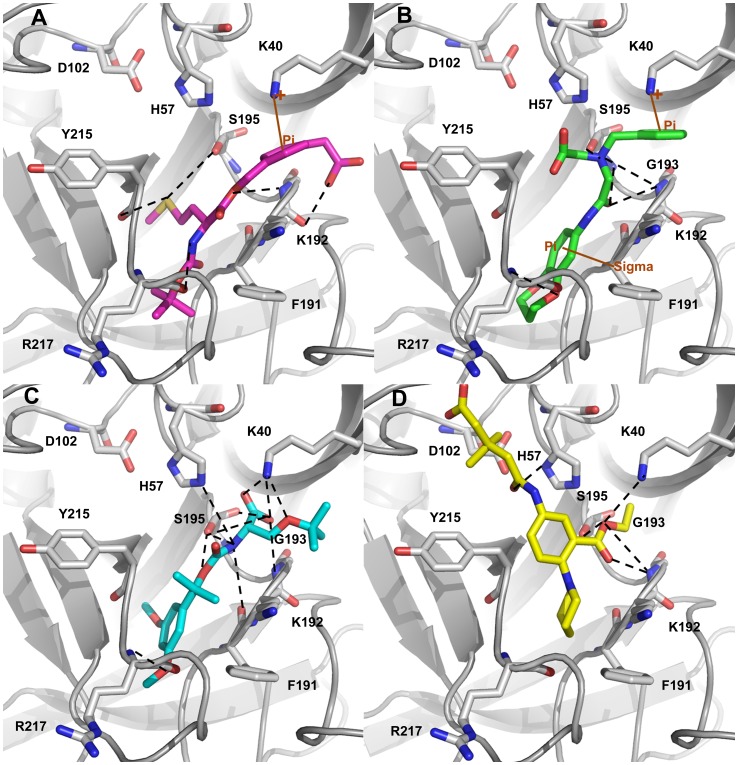
Molecular docking results. The binding modes of (A) Hit 1 (B) Hit 2 (C) Hit 3 and (D) Hit 4 at the active site of the enzyme. The amino acid residues and bound ligands are shown in stick representations. The hydrogen bond, and π-interactions are shown in black dashed, and brown solid lines. Only polar hydrogen atoms are added for clarity.

The binding mode of Hit 1 within the active site of chymase has been analyzed. This compound was identified from Maybridge database. The GOLD Fitness score and Autodock binding free energy values for Hit 1 were 72.018 and −6.42 kcal/mol, respectively. This hit compound has shown a different binding mode compared to that of C1, the most active compound, but bounds very tightly with the catalytic components. As shown in the molecular docking result, the suitable shape of this hit compound helped it to bind tightly with the binding pocket of chymase, facilitating π^…^cationic interactions between K40 and aromatic ring of Hit 1(Figure 13A). Moreover, a network of hydrogen bonds between Hit 1 and key amino acids including K192(2.971 Å), G193(2.674 Å), S195 (3.031 Å), Y215(2.825 Å), and R217(3.047 Å) of binding site cleft was also formed, which suggested that Hit 1could be a potent inhibitor of human chymase.

### Binding Mode of Hit 2

Hit 2 had a GOLD Fitness score of 70.268 and an Autodock binding free energy of –5.72 kcal/mol. The binding mode of Hit 2 is similar to Hit 1. Upon the examination of docking features between Hit 2 and chymase it was found that various kinds of close contacts which include π^…^cationic, π^…^σ, hydrophobic and hydrogen bonding were formed between the hit compound and active site region of enzyme. The π^…^cationic interaction was observed between positively charged residue K40 and aromatic ring of Hit 2 as depicted in Figure 13B. A π^…^σ interaction was also found between the hydrophobic residue F191 and benzodioxepine ring of Hit 2. Furthermore, a number of hydrogen bonds were formed between K192, G193, S195, and R217 of active site and Hit 2compound.

### Binding Mode of Hit 3

The binding conformation of Hit 3 within the active site of chymase had a GOLD Fitness score of 70.107 and an Autodock binding free energy of −6.21 kcal/mol. It also bonded to the active site in a similar fashion to the other two hit compounds. Hit 3 formed a number of hydrogen bonds with the catalytically important residues like K40(2.019, 2.011, and 3.0 Å), K192(2.616 Å), G193(3.124 Å), S195(3.110 Å), and R217(3.124 Å). It was also able to establish hydrophobic interaction with F191 of binding site cleft. Due to its tight binding to active site of enzyme, and presence of key interactions, Hit 3 could be a potent inhibitor of chymase enzyme.

### Binding Mode of Hit 4

The binding mode of this hit compound is similar to C2 and C3 compounds studied in this research exertion. The molecular interactions exhibited by this hit compound included the hydrogen bond interactions with K40 (2.628 Å), H57 (2.933 Å), G193(2.680 Å), (3.183 Å), and S195(3.294Å) (Figure 13D). The GOLD Fitness score and Autodock binding free energy values for Hit 4 were 68.195 and −5.11 kcal/mol, respectively. The lower energy gap value was also promising for this compound which further enhanced its constancy to be apotential lead compound.

A search using SciFinder Scholar and PubChem Structure search tools has also confirmed that the identified hits were not reported elsewhere earlier for the inhibition of human chymase.

### Conclusions

Chymase is an enzyme of the hydrolase class that catalyzes the hydrolysis of peptide bonds and it is the major extravascular source of vasoactive Ang II, which is generated with exceptional efficiency by human chymase via hydrolysis of the Phe-8–His-9 bond of Ang I. The inhibition of chymase is likely to divulge therapeutic ways for the treatment of cardiovascular diseases, allergic inflammation, and fibrotic disorders. Therefore, several peptide and non-peptide inhibitors of chymase have been synthesized by various research groups. We selected three experimentally known chymase inhibitors which have very similar chemical structures but exhibited the considerable differences in terms of chymase inhibitory activity with the aim of finding the reasons for the difference observed in their inhibitory profiles caused by the subtle structural variation. The disparities in hydrogen bond networks and non-bonded interaction energies between the inhibitor and catalytic residues along with binding conformations of the inhibitors have explained the difference in their biological activities very convincingly. The evaluation of hydrogen bond network has demonstrated that the most active compound has formed the maximum number of short-ranged hydrogen bond interactions with the positively charged K40 residue at the active site. This residue was reported to hold an important role in the substrate recognition and hydrolysis process. Thus, formation of strong interactions with important catalytic K40 residue by an inhibitor is considered very significant for effective inhibitor binding. The key structural changes comprise of the secondary structural changes spotted at the regions of V36-F41, and G216-A220 residues that determine the conformation and flexibility of very important catalytic K40, and R217 residues. Interestingly, the region around K40 has exhibited less flexibility in substrate as well asmost active C1 and less active C2 complexes whereas this region has shown maximum flexibility in the least active C3 complex. In the G216-A220 region, all of these five residues were present in variable regions thus forming a helix in the least active C3 complex. However, this loop region showed more rigidity in the case of substrate and less active C2 complexes while two residues of G216-A220 region were in variable regions in C1complex. These appreciable distinctions in binding modes, structural differences, and key interactions determined the basis for the potent inhibitory profile of C1, slightly reduced activity of C2, and the least active nature of C3. The dynamic structures of chymase-C1 and chymase-Ang I complexes were employed in the development of a hybrid pharmacophore model which was subjected to screening of the druglike databases to select the compounds containing all the hybrid pharmacophoric features identified from both the most active inhibitor and substrate. The compounds that resulted from the pharmacophore filtering were docked into the active site of human chymase and the hits those bind well at the active site, exhibited key interactions and favorable electronic properties were identified as possible inhibitors for chymase enzyme. Finally four compounds of diverse chemical scaffolds were selected as potential leads to be used in novel and potent chymase inhibitor design. This study not only explains the inhibitory mechanism of the inhibitors but also provides important structural insights which broads our knowledge for drug designing against enzyme targets.
